# Inhibition of proinflammatory signaling impairs fibrosis of bone marrow mesenchymal stromal cells in myeloproliferative neoplasms

**DOI:** 10.1038/s12276-022-00742-y

**Published:** 2022-03-14

**Authors:** Milica Vukotić, Sunčica Kapor, Teodora Dragojević, Dragoslava Đikić, Olivera Mitrović Ajtić, Miloš Diklić, Tijana Subotički, Emilija Živković, Bojana Beleslin Čokić, Aleksandar Vojvodić, Juan F. Santibáñez, Mirjana Gotić, Vladan P. Čokić

**Affiliations:** 1 Institute for Medical Research, National Institute of Republic of Serbia, University of Belgrade, Belgrade, Serbia; 2 Department of Hematology, Clinical Hospital Centre Dragisa Mišović, Belgrade, Serbia; 3 Clinic for Endocrinology, Diabetes and Metabolic Diseases Genetic Laboratory, University Clinical Center of Serbia, Belgrade, Serbia; 4 Department of Orthopedic Surgery, Clinical Center Zemun, Belgrade, Serbia; 5 Centro Integrativo de Biología y Química Aplicada (CIBQA), Universidad Bernardo O’Higgins, Santiago, Chile; 6 Clinic of Hematology, University Clinical Center of Serbia, Belgrade, Serbia; 7 Medical Faculty, University of Belgrade, Belgrade, Serbia

**Keywords:** Mesenchymal stem cells, Chemotherapy

## Abstract

Although bone marrow-derived mesenchymal stromal cells (BM-MSCs) have been identified as a major cellular source of fibrosis, the exact molecular mechanism and signaling pathways involved have not been identified thus far. Here, we show that BM-MSCs contribute to fibrosis in myeloproliferative neoplasms (MPNs) by differentiating into αSMA-positive myofibroblasts. These cells display a dysregulated extracellular matrix with increased FN1 production and secretion of profibrotic MMP9 compared to healthy donor cells. Fibrogenic TGFβ and inflammatory JAK2/STAT3 and NFκB signaling pathway activity is increased in BM-MSCs of MPN patients. Moreover, coculture with mononuclear cells from MPN patients was sufficient to induce fibrosis in healthy BM-MSCs. Inhibition of JAK1/2, SMAD3 or NFκB significantly reduced the fibrotic phenotype of MPN BM-MSCs and was able to prevent the development of fibrosis induced by coculture of healthy BM-MSCs and MPN mononuclear cells with overly active JAK/STAT signaling, underlining their involvement in fibrosis. Combined treatment with JAK1/2 and SMAD3 inhibitors showed synergistic and the most favorable effects on αSMA and FN1 expression in BM-MSCs. These results support the combined inhibition of TGFβ and inflammatory signaling to extenuate fibrosis in MPN.

## Bone marrow disorders: Combined treatment option for fibrosis

The treatment of fibrosis in patients with rare bone marrow disorders could be improved with a combined therapy that targets inflammatory pathways. Myeloproliferative neoplasms (MPN) are a group of bone marrow disorders characterized by the over-production of blood cells, which can lead to fibrosis in the bone marrow. Vladan Čokić at the University of Belgrade, Serbia, and co-workers examined how stem cells known as mesenchymal stromal cells from the bone marrow contribute to MPN fibrosis. They found an increase in three pro-inflammatory signaling pathways in MPN patients, resulting in the stromal cells differentiating into cells with dysregulated extracellular matrices. The differentiated cells did not behave correctly nor degrade properly, triggering fibrosis. The team combined two drugs that target the inflammatory signaling pathways, and successfully inhibited the development of fibrosis in MPN cell cultures.

## Introduction

Myeloproliferative neoplasms (MPNs) are clonal hematopoietic disorders characterized by an overproduction of erythrocytes, thrombocytes and/or leukocytes driven by mutations in Janus kinase 2 (JAK2), calreticulin, or myeloproliferative leukemia-related protein-encoding genes that induce constitutive activation of the Janus kinase-signal transducer and activator of transcription (JAK-STAT) signaling pathway^1,2^. Philadelphia-negative MPNs include polycythemia vera (PV), essential thrombocythemia (ET), and primary myelofibrosis (PMF)^3^. Bone marrow fibrosis (BMF) is the most pronounced in PMF and represents the major diagnostic criterion^4^. Nevertheless, the development of fibrosis is a common feature of all MPNs^5^. Studies suggest a correlation between the grade of BMF and the prognosis of MPNs, with more fibrosis associated with a poorer outcome^6,7^. The only intervention considered to be curative in myelofibrosis is allogeneic hematopoietic stem cell transplantation, but high treatment-related morbidity and mortality limit its use^8^. The JAK1/2 inhibitor ruxolitinib provides only a limited reduction in fibrosis^9^, highlighting the need to target specific cells and pathways involved in fibrosis development and progression.

Fibrosis is a pathophysiological process characterized by aberrant myofibroblast accumulation and excessive deposition of reticulin and collagen fibers, as well as other components of the extracellular matrix (ECM)^4^. It has been suggested that BMF in MPNs occurs due to aberrant growth factor and cytokine secretion from mutated hematopoietic progenitors^10^. Transforming growth factor beta (TGFβ), platelet-derived growth factor, and fibroblast growth factor affect surrounding cells in the bone marrow niche, leading to fibrosis^5^. Bone marrow myofibroblasts are a highly heterogeneous population originating from mesenchymal stromal cells^11,12^, pericytes, monocytes^13,14^, and even adipocytes^15,16^. However, perivascular and endosteal glioma-associated oncogene homolog 1 (Gli1)-positive bone marrow-derived mesenchymal stromal cells (BM-MSCs) have recently been identified as a major cellular origin of BMF and a relevant therapeutic target^17^.

In this study, we investigated the fibrotic phenotype of BM-MSCs isolated from MPN patients, as well as the involvement of inflammation and the TGFβ/mothers against decapentaplegic homolog 3 (SMAD3) and JAK2/STAT3 signaling pathways. Fibrosis was assessed by analyzing the expression of the myofibrotic marker alpha smooth muscle actin (αSMA), production of fibronectin (FN1), and secretion of matrix metalloproteinases (MMPs) 2 and 9. Furthermore, we analyzed whether treatment of MPN-derived BM-MSCs with the JAK1/2 inhibitor ruxolitinib, SMAD3 inhibitor SIS3 and nuclear factor kappa-light-chain-enhancer of activated B cells (NFκB) inhibitor JSH23 or their combination could diminish the fibrotic phenotype. Investigating the cellular origin of molecular pathways that contribute to fibrosis in MPN is of interest for developing targeted antifibrotic treatments.

## Materials and methods

### Patient characteristics

BM-MSCs were isolated from 27 MPN patients and 8 healthy donors. Out of the 27 patients, 11 were classified as having PV, 7 were classified as having ET, and 9 were classified as having PMF. Twenty-five patients were positive for the *JAK2* V617F mutation, whereas 2 did not have the *JAK2* V617F mutation. The median age of the patients, comprising 15 males and 12 females, was 66.74 years (range: 31–88 years) at the time of diagnosis, while the median age of the healthy controls, comprising 5 males and 3 females, was 51.74 years (range: 29–78 years). The grade of fibrosis was assessed at the time of diagnosis by staining reticulin fibers on bone marrow biopsies based on Gomori silver impregnation. Reticulin staining was graded as follows: 0 (absent), 1 (fine fibers), 2 (diffuse fine fiber network), and 3 (diffuse fiber network with scattered coarse fibers). PB-MNCs were isolated from 15 patients and 3 healthy donors; 3 patients with PV, 3 patients with ET, and 3 patients with PMF had the *JAK2* V617F mutation, while 6 patients were *JAK2* mutation-negative (3 with ET and 3 with PMF). Patients were diagnosed with MPN according to the World Health Organization (WHO) 2016 classification. Informed consent was obtained from all of the participants included in the study, which was approved by the local Ethical Committee.

### BM-MSC and PB-MNC cocultures

BM-MSCs (1 × 10^5^ cells/well) were seeded on glass coverslips in 24-well plates (experiments for each donor were performed in triplicate). After adherence of the BM-MSCs, PB-MNC suspensions (2.5 × 10^5^ and 5 × 10^5^ cells/well) were added to the culture in a final volume of 300 μl/well of RPMI-1640 medium (Biowest, Nuaillé, France) supplemented with 10% FBS, (100 units/ml, Biowest), Pen-Strep (Biowest) and amphotericin B (Biowest). This coculture was maintained for 7 days at 37 °C and 5% CO_2_.

### Immunofluorescence

Briefly, 3 × 10^4^ cells were seeded on rounded cover slips in 24-well plates and treated the next day as indicated. Cell monolayers were fixed with 4% paraformaldehyde in PBS and permeabilized with 0.2% Triton X-100 (Sigma-Aldrich, St. Louis, MO, USA) for 15 min. Blocking was performed for 2 h at room temperature with 1% BSA in PBS with 0.05% Tween 20 (PBST). Cells were incubated with primary antibodies overnight at 4 °C and washed 3 times for 5 min with 0.1% PBST. Primary antibodies were directed against αSMA (Abcam, Cambridge ab7817, UK), FN1 (Sigma Aldrich, F7387), and pNFκB Ser536 (Cell Signaling, Danvers, MA 3031, USA). Secondary antibodies were conjugated with AlexaFlour-488 and AlexaFlour-547 fluorescent dyes (Thermo Fisher Scientific, Waltham, MA, USA, A-11034 and A-11029, respectively) and incubated with the cells for 1 h at room temperature. Nuclei were counterstained with DAPI (Sigma Aldrich, D-9542). After mounting with DABCO-Mowiol, the samples were examined and photographed using an epifluorescence microscope (Olympus Provis AX70, Tokyo, Japan). Quantification of αSMA-positive cells and the FN1 fluorescence area was performed in ImageJ. The percentage of positive cells and FN1 fluorescence were calculated based on the total number of DAPI-positive nuclei and the total image area, respectively. An average of four sections per patient were quantified.

### Zymography assay

MMP2 and MMP9 secretion was examined as described previously by Santibanez et al. (2002)^18^. Briefly, 3 × 10^4^ cells were seeded in 24-well plates and cultured overnight. Then, the cells were washed with PBS and cultured in serum-free culture medium with appropriate treatments. Protein concentration was determined from in the cell lysate by a BCA Protein Assay Macro Kit (Serva, Heidelberg, Germany) according to the manufacturer’s instructions. Medium with secreted proteins was collected and subjected to 8% SDS–PAGE in 0.1% gelatin gels under nonreducing conditions. The gels were washed twice with 2.5% Triton X-100 for 30 min and incubated for 24 h in 100 mM Tris–HCl, pH 8.5, with 10 mM CaCl_2_ to allow folding and activation of MMP enzymes. The activity of MMPs was stopped by staining the gels with Coomassie Blue R250 (Thermo Fisher Scientific) in 50% methanol and 10% acetic acid for 10 min. The gels were distained in 20% methanol and 5% acetic acid until transparent bands were observed. Imaging and quantification of bands were performed using ChemiDoc Imager (Bio-Rad Laboratories, Hercules, CA, USA) and ImageLab software (v. 6.0.0.25, Bio-Rad, Hercules, USA).

## Results

### Fibrosis in bone marrow-derived mesenchymal stromal cells of MPN patients

**Fig. 1:**
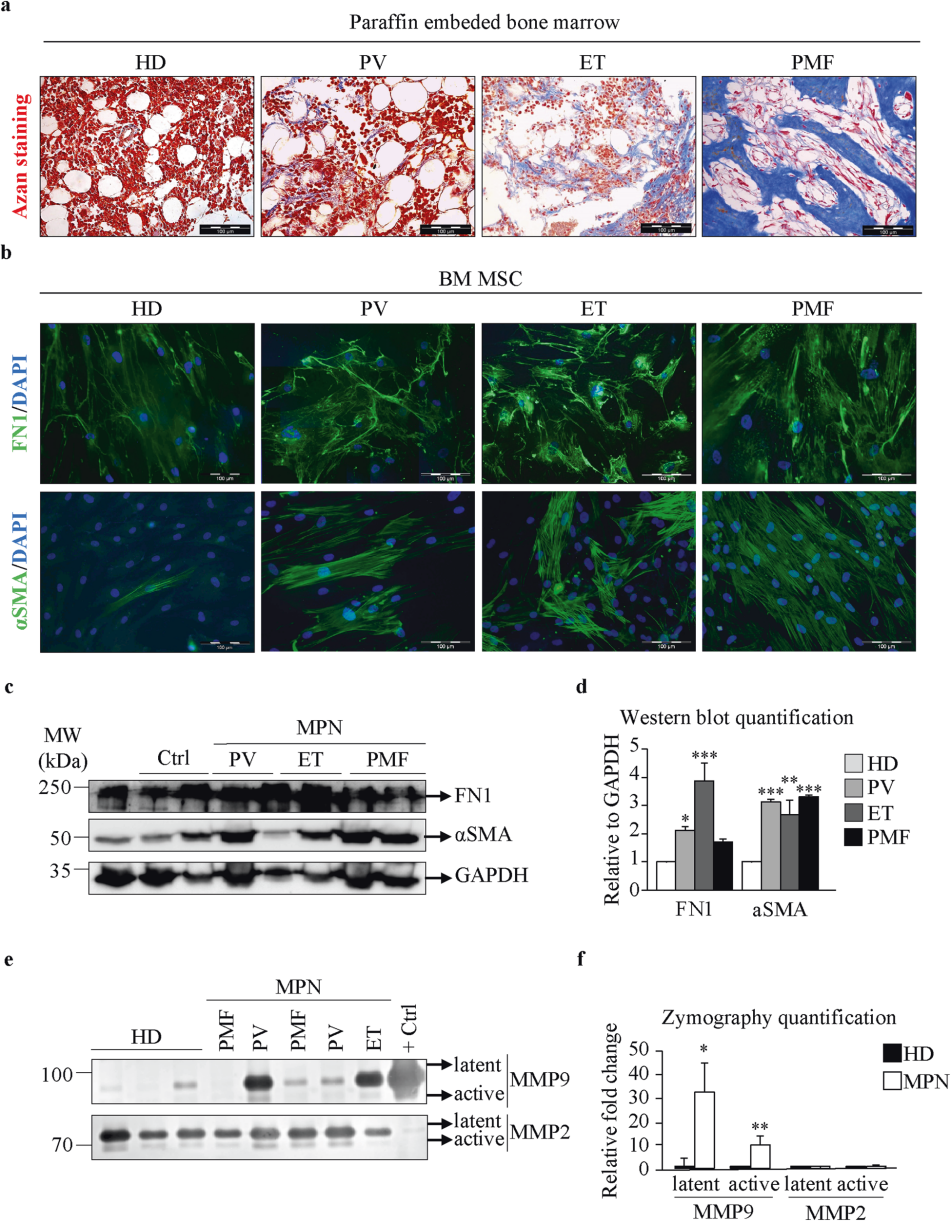
Bone marrow mesenchymal stromal cells from MPN patients display a fibrotic phenotype. **a** Azan staining of paraffin-embedded bone marrow samples from healthy donors (HDs) and patients with polycythemia vera (PV), essential thrombocythemia (ET), and primary myelofibrosis (PMF). **b** Immunofluorescence assay of bone marrow mesenchymal stromal cells (BM-MSCs) isolated from HDs and PV, ET, and PMF patients. Fibronectin (FN1) and alpha smooth muscle actin (αSMA) are shown in green. Nuclei were counterstained with 4ʹ,6 diamidino-2-phenylindole (DAPI, blue). Scale bar 100 µm. **c** Western blot indicating the protein expression of FN1 and αSMA. Glyceraldehyde 3-phosphate dehydrogenase (GAPDH) was used as a loading control. **d** Quantification of the FN1 and αSMA Western blot bands. **e** Zymography assay showing the levels of the latent and active forms of secreted matrix metalloproteinases 2 and 9 (MMP2 and MMP9). **f** Quantification of the MMP2 and MMP9 zymography assay bands. *n* = 4; **d** and **f** mean + SEM, *p < 0.05, **p < 0.01, ***p < 0.001.

Fibrosis was studied in 27 total patients with MPNs, with clinical and laboratory characteristics presented in Supplementary Fig. 1a. To assess BMF, we stained paraffin-embedded bone marrow samples isolated from PV, ET, and PMF patients and healthy donors (HD) with Azan Trichrome staining, for which blue dye indicates collagen deposition. Indeed, PMF patients showed prominent central interconnected fibers with extensive collagen staining throughout the tissue (Fig. 1a). Low levels of collagen deposition were observed in PV patients, with mostly perivascular localization, whereas ET patients showed moderate collagen accumulation (Fig. 1a). Since BM-MSCs have been identified as a major cellular source of BMF^17^, we wanted to test whether BM-MSCs from MPN patients display a myofibrotic phenotype. BM-MSCs were isolated by preplating, and flow cytometry confirmed the expression of the mesenchymal stromal surface markers CD73, CD90, and CD105 and negativity for the leukocyte markers CD11b and CD45 (Supplementary Fig. 1b). Isolated BM-MSCs exhibited a stellate fibroblast-like morphology and possessed the potential to differentiate into adipocytes and osteoblasts when exposed to specific media, as shown by Oil Red O and Alizarin Red S staining, respectively (Supplementary Fig. 1c). BM-MSC fibrosis was assessed by determining the expression of the myofibroblast marker αSMA and the production of FN1, as an early fibrosis marker and ECM component^19^, by immunofluorescence and Western blot. BM-MSCs from MPN patients showed high αSMA positivity and a significant increase in FN1 expression compared to those from healthy donors (Fig. 1b–d). Since MMPs can modulate the ECM to exert both profibrotic and antifibrotic functions^20^, we proceeded to investigate the secretion of precleaved latent and catalytically active MMP2 and MMP9 from BM-MSCs by zymography assays. Zymography assays revealed that secretion of the active and latent forms of MMP2 did not change, whereas MMP9 production was significantly increased in MPN patients compared to HDs (Fig. 1e, f). In summary, BM-MSCs isolated from all three types of MPN had a myofibrotic phenotype characterized by high intracellular αSMA positivity, increased production and expression of FN1, and increased extracellular profibrotic MMP9 levels.

### TGFβ/SMAD3 and JAK2/STAT3 signaling in bone marrow-derived mesenchymal stromal cells from MPN patients

**Fig. 2:**
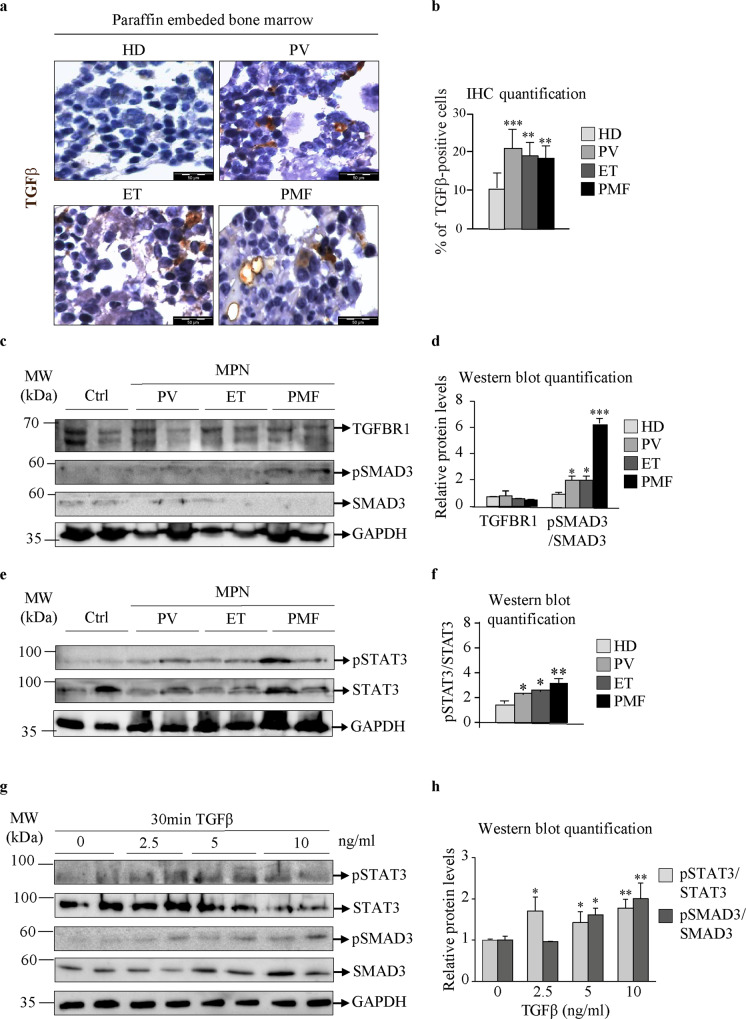
TGFβ and JAK2/STAT3 signaling in bone marrow mesenchymal stromal cells from MPN patients. Paraffin-embedded bone marrow samples from healthy donors (HDs) and patients with polycythemia vera (PV), essential thrombocythemia (ET) and primary myelofibrosis (PMF). **a** Immunocytochemistry assay for transforming growth factor beta (TGFβ; shown in brown). Nuclei were counterstained with hematoxylin (purple). Scale bar 50 µm. **b** Quantification of immunocytochemistry results, representing the percentage of TGFβ-positive cells. **c** Bone marrow mesenchymal stromal cells (BM-MSCs) from HDs and patients with PV, ET, and PMF. Western blot indicating the protein expression of TGFβ receptor 1 (TGFBR1), phosphorylated mothers against decapentaplegic homolog 3 (pSMAD3), and total SMAD3. **d** Quantification of the TGFBR1 and pSMAD3 Western blot bands. **e** Western blot indicating the protein expression of phosphorylated signal transducer and activator of transcription 3 (pSTAT3) and total STAT3. Glyceraldehyde 3-phosphate dehydrogenase (GAPDH) was used as a loading control. **f** Quantification of the pSTAT3 and STAT3 Western blot bands. **g** HD BM-MSCs treated with the indicated concentrations of TGFβ for 30 min. Western blot indicating the protein expression of STAT3, pSTAT3, SMAD3, and pSMAD3. GAPDH was used as a loading control. **h** Quantification of the STAT3 and SMAD3 Western blot bands. *n* = 4; **b**, **d**, **f**, and **h** mean + SEM, *p < 0.05, **p < 0.01, ***p < 0.001.

Previous studies have shown that the expression and production of TGFβ are increased in patients suffering from MPNs^21^. These findings were confirmed in our paraffin-embedded bone marrow samples by immunohistochemistry (Fig. 2a, b). In addition, microarray data from CD34-positive cells isolated from peripheral blood showed increased levels of TGFβ and TGFβ receptor 1 (TGFBR1) mRNA in MPN patients compared to HDs^22^ (Supplementary Fig. 2a). We next analyzed the expression of TGFBR1 and phosphorylated SMAD3 (pSMAD3) proteins to determine whether MPN BM-MSCs respond to the increased TGFβ levels in their niche. We did not find any differences in the protein levels of TGFBR1 between MPN and HD BM-MSCs (Fig. 2c, d). However, the expression of pSMAD3 was significantly increased in all MPN entities, most prominently in PMF (Fig. 2c, d), indicating that patients with MPNs showed increased TGFβ/SMAD3 signaling compared to HDs. Furthermore, in MPN BM-MSCs, we detected increased levels of pSTAT3 protein, indicating activation of the JAK/STAT signaling pathway (Fig. 2e, f). These results were corroborated by the in vitro induction of fibrosis in healthy BM-MSCs treated with TGFβ (Supplementary Fig. 2b–d). TGFβ signaling led to an increase in pSMAD3 and pSTAT3 proteins, indicating convergence of the TGFβ/SMAD3 and JAK2-STAT3 signaling pathways (Fig. 2g, h). Together, these data indicate that TGFβ signaling induces a fibrotic phenotype in BM-MSCs and leads to STAT3 phosphorylation and activation.

### The effect of JAK1/2 inhibition on TGFβ-induced fibrosis in bone marrow mesenchymal stromal cells

**Fig. 3:**
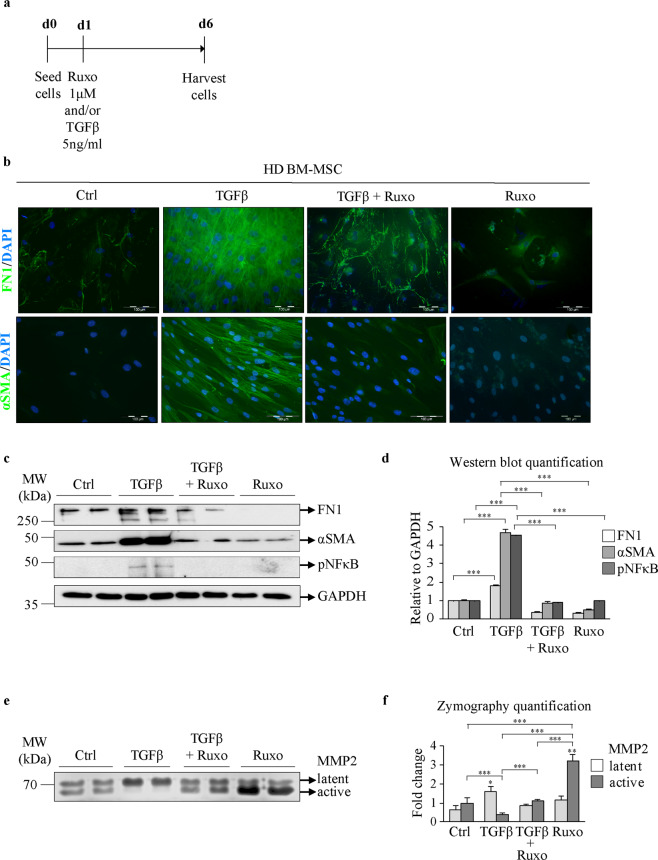
Treatment with ruxolitinib decreases TGFβ-induced fibrosis in mesenchymal stromal cells. **a** Bone marrow mesenchymal stromal cells from healthy donors (HD BM-MSCs) were treated with transforming growth factor beta (TGFβ), ruxolitinib (Ruxo) or their combination as indicated. **b** Immunofluorescence assay for fibronectin (FN1) and alpha smooth muscle actin (αSMA), with staining shown in green. Nuclei were counterstained with 4ʹ,6 diamidino-2-phenylindole (DAPI, blue). Scale bar 100 µm. **c** Western blot indicating the protein expression of FN1, αSMA, and phosphorylated nuclear factor kappa-light-chain-enhancer of activated B cells (pNFκB). Glyceraldehyde 3-phosphate dehydrogenase (GAPDH) was used as a loading control. **d** Quantification of the FN1, αSMA, and pNFκB Western blot bands. **e** Zymography assay showing the levels of the latent and active forms of secreted matrix metalloproteinase 2 (MMP2). **f** Quantification of the MMP2 zymography assay bands. *n* = 4; **d** and **f** mean + SEM, *p < 0.05, **p < 0.01, ***p < 0.001.

To test whether blockade of JAK-STAT signaling can impair the TGFβ-induced fibrosis mitigated by BM-MSCs, we treated these cells with the JAK1/2 inhibitor ruxolitinib (Ruxo), as depicted in Fig. 3a. A TGFβ concentration of 5 ng/ml was chosen since it was found to be sufficient to induce fibrosis (Fig. 3b–d) but did not have a cytotoxic effect on BM-MSCs (Supplementary Fig. 3a). The concentration of 1 µM Ruxo also did not cause cytotoxicity (Supplementary Fig. 3b) and represents the clinically observed plasma peak level^23^. Cotreatment of HD BM-MSCs with TGFβ and Ruxo markedly decreased the expression of αSMA and FN1 (Fig. 3b–d) compared to treatment with TGFβ alone, confirming the involvement of JAK-STAT signaling in inducing the myofibrotic phenotype. TGFβ treatment induced pNFκB expression, which was abrogated by Ruxo cotreatment (Fig. 3c, d). Zymography assays showed that control BM-MSCs produced both active and latent forms of MMP2 to a similar extent (Fig. 3e, f). TGFβ treatment abolished the secretion of the active form of MMP2, which was rescued by Ruxo (Fig. 3e, f). These data indicate that JAK1/2 signaling mediates TGFβ-induced fibrosis in BM-MSCs.

### Inflammatory IL6 signaling contributes to TGFβ-induced fibrosis of mesenchymal stromal cells

**Fig. 4:**
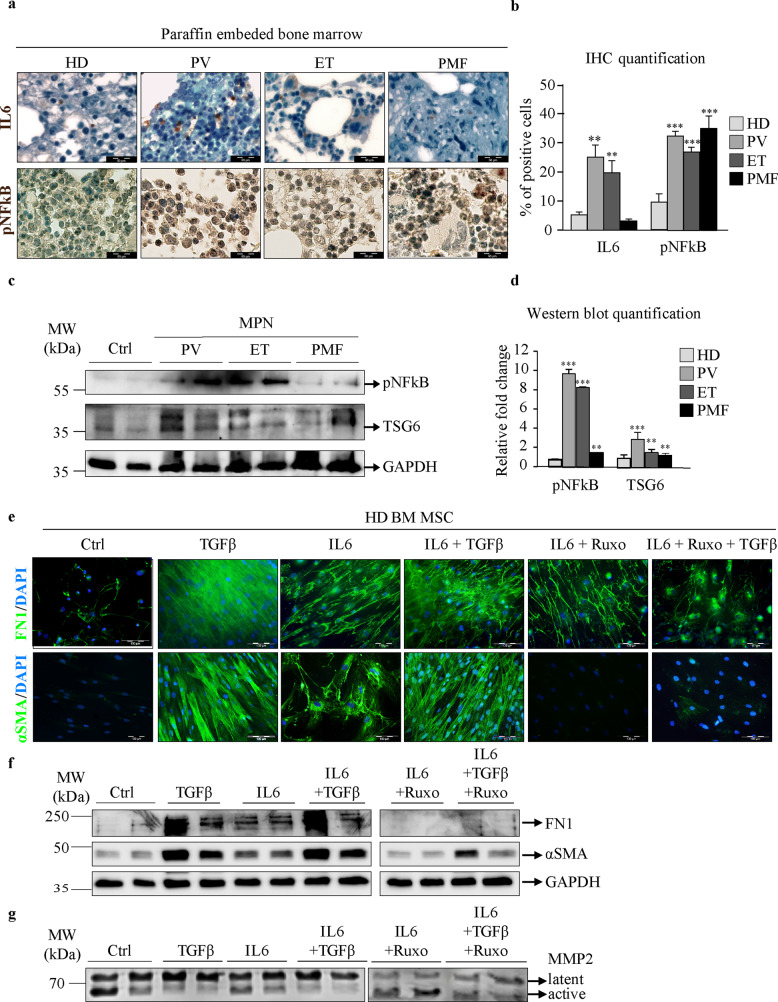
Inflammatory IL6 signaling contributes to TGFβ-induced fibrosis in mesenchymal stromal cells. Paraffin-embedded bone marrow samples from healthy donors (HDs) and patients with polycythemia vera (PV), essential thrombocythemia (ET) and primary myelofibrosis (PMF). **a** Immunocytochemistry assay for interleukin 6 (IL6) and phosphorylated NFκB (pNFκB), with staining shown in brown. Nuclei were counterstained with hematoxylin (blue). Scale bar 50 µm. **b** Quantification of the immunocytochemistry staining representing the percentage of IL6- or pNFκB-positive cells. **c** Bone marrow mesenchymal stromal cells (BM-MSCs) from HDs and patients with PV, ET, and PMF. Western blot indicating the protein expression of phosphorylated nuclear factor kappa-light-chain-enhancer of activated B cells (pNFκB) and tumor necrosis factor-stimulated gene 6 (TSG6). Glyceraldehyde 3-phosphate dehydrogenase (GAPDH) was used as a loading control. **d** Quantification of the pNFκB and TSG6 Western blot bands. **e** HD BM-MSCs were treated with IL6, transforming growth factor beta (TGFβ) and/or ruxolitinib (Ruxo). Immunofluorescence assays for fibronectin (FN1) and alpha smooth muscle actin (αSMA) were performed, and staining is shown in green. Nuclei were counterstained with 4ʹ,6 diamidino-2-phenylindole (DAPI, blue). Scale bar 100 µm. **f** Western blot indicating the protein expression of FN1 and αSMA. GAPDH was used as a loading control. **g** Zymography assay showing the levels of the latent and active forms of secreted matrix metalloproteinase 2 (MMP2). *n* = 4; **b** and **d** mean + SEM, *p < 0.05, **p < 0.01, ***p < 0.001.

Since the JAK/STAT signaling pathway is known to be activated by proinflammatory cytokines secreted by BM-MSCs, we next proceeded to investigate whether inflammation in MPNs contributed to the fibrosis induced by BM-MSCs. To assess inflammation in bone marrow samples from MPN patients, we determined the expression of proinflammatory interleukin 6 (IL6) and phosphorylated NFκB (pNFκB) by immunocytochemistry. IL6 expression was highly increased in PV and ET patient bone marrow aspirates, whereas the number of cells expressing pNFκB was increased in all three MPN entities compared to that in HDs (Fig. 4a, b). These results are in accordance with the increased JAK/STAT signaling detected in BM-MSCs, indicating an increased inflammatory response.

Furthermore, to investigate the inflammatory response in BM-MSCs, we analyzed the expression of pNFκB and tumor necrosis factor-stimulated gene 6 (TSG6) by Western blot. NFκB signaling, a key regulator of hepatic myofibroblast activation^24^, was found to be active in MPN BM-MSCs, since the expression of pNFκB subunit p65 was significantly increased in patients compared to HDs (Fig. 4c, d). These findings are in accordance with the levels of nuclear pNFκB in MPN BM-MSCs detected by immunofluorescence, which were not observed in HD cells (Supplementary Fig. 4a). Moreover, activated NFκB signaling was present in MPN BM-MSCs despite slightly increased expression of the anti-inflammatory mediator TSG6 (Fig. 4c, d).

To investigate whether proinflammatory JAK2-STAT3 signaling contributes to the TGFβ-induced fibrotic phenotype of BM-MSCs, we treated the cells with proinflammatory interleukin 6 (IL6) and/or Ruxo for 6 days as depicted in Supplementary Fig. 4b. IL6 treatment induced the JAK/STAT signaling pathway in a time-dependent manner, as evidenced by increased protein expression of pSTAT3 (Supplementary Fig. 4c, d). IL6 and TGFβ mutually increased FN1 and αSMA levels (Fig. 4e, f and Supplementary Fig. 4e). In accordance with previous results, cotreatment with Ruxo led to a decrease in αSMA and FN1, regardless of TGFβ (Fig. 4e, f and Supplementary Fig. 4e). Furthermore, IL6 induced an increase in the nuclear pNFκB signal detected by immunofluorescence, which was further potentiated by TGFβ (Supplementary Fig. 4g). The secretion of active MMP2 was slightly decreased after IL6 treatment, although this change was not as affected by TGFβ (Fig. 4g and Supplementary Fig. 4f). The change was abolished with the addition of Ruxo (Fig. 4g and Supplementary Fig. 4f). Taken together, our results indicate that inflammation induced by IL6 has a major effect on the fibrotic phenotype of BM-MSCs by affecting αSMA and FN1 levels and decreasing the secretion of MMP2.

### Impact of MPN mononuclear cells on the fibrosis induced by BM-MSCs

**Fig. 5:**
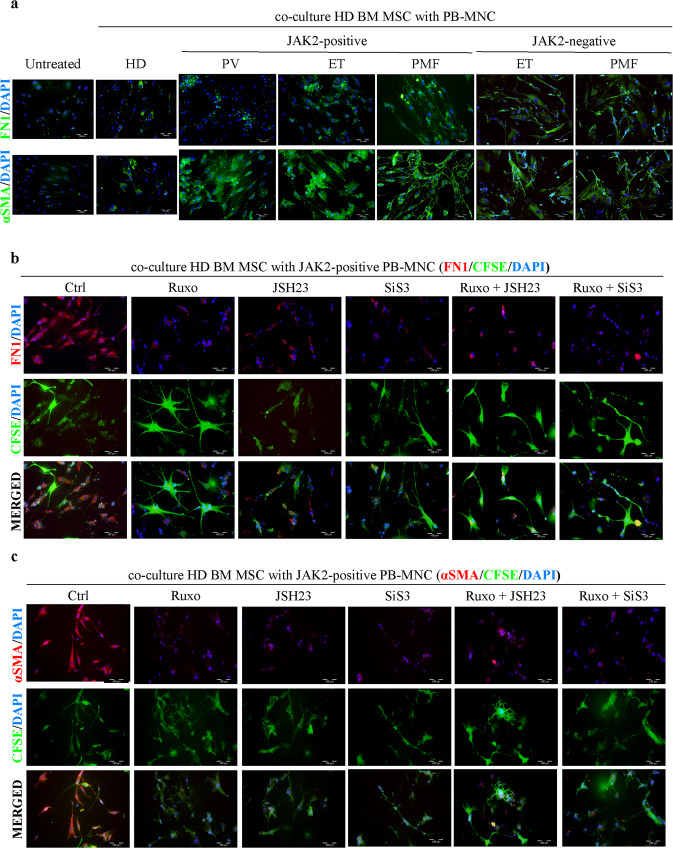
MPN mononuclear cells induce fibrosis via mesenchymal stromal cells. Immunofluorescence assay of (**a**) healthy bone marrow mesenchymal stromal cells (BM-MSCs) incubated with *JAK2* V617F-positive or -negative peripheral blood mononuclear cells (PB-MNCs) isolated from patients with polycythemia vera (PV), essential thrombocythemia (ET) and primary myelofibrosis (PMF) or with PB-MNCs from healthy donors (HDs). Immunofluorescence staining of fibronectin (FN1) and alpha smooth muscle actin (αSMA) is shown in green. Nuclei were counterstained with 4ʹ,6 diamidino-2-phenylindole (DAPI, blue). Scale bar 100 µm. Healthy BM-MSCs were incubated with PB-MNCs isolated from *JAK2* V617F-positive PMF patients and treated with specific SMAD3 (SIS3), NFκB (JSH23) and JAK1/2 (Ruxo) inhibitors alone or in combination for 48 h. Immunofluorescence staining of (**b**) FN1 and (**c**) αSMA is shown in red. The vital dye carboxyfluorescein succinimidyl ester (CFSE) is shown in green. Nuclei are counterstained with DAPI (blue). Scale bar 100 µm. *n* = 3.

Since BM-MSCs are not part of the neoplastic clone in MPNs^25^, we wanted to investigate what triggers the fibrotic phenotype in BM-MSCs. To test whether BM-MSCs are instructed to develop a myofibrotic phenotype by mutated MPN hematopoietic cells, we performed coculture experiments of peripheral blood-derived mononuclear cells (PB-MNCs) isolated from MPN patients with the *JAK2* V617F mutation with BM-MSCs isolated from healthy donors. PB-MNCs from healthy donors were used as *JAK2* V617F-negative controls. After 48 h, the potential of HD BM-MSCs to induce fibrosis was assessed by measuring FN1 and αSMA expression by immunofluorescence. PB-MNCs from PMF and, to a lesser extent, ET patients were able to induce FN1 production in BM-MSCs, unlike PV PB-MNCs (Fig. 5a and Supplementary Fig. 5a). However, HD BM-MSCs showed high αSMA positivity after incubation with PB-MNCs from all three diseases (Fig. 5a and Supplementary Fig. 5a). Interestingly, the fibrotic phenotype got stronger with disease progression (PV < ET < PMF), with the most striking phenotypic change induced by PB-MNCs from PMF patients. Incubation with *JAK2* V617F-negative PB-MNCs from ET and PMF patients was also able to induce expression of profibrotic markers in healthy BM-MSCs—FN1 was expressed to a similar level, while αSMA expression was somewhat lower than that seen in the coculture with *JAK2* V617F-positive PB-MNCs but was significantly increased compared to that in HD PB-MNC (Fig. 5a and Supplementary Fig. 5a). These results indicate that excessive activation of JAK/STAT signaling in PB-MNCs is sufficient to induce a profibrotic phenotype of BM-MSCs, regardless of their mutational status.

Moreover, we wanted to test whether fibrotic BM-MSCs from MPN patients secrete factors that further support fibrosis. To this end, we incubated HD BM-MSCs with medium collected from MPN BM-MSCs for 48 h and monitored FN1 and αSMA expression by immunofluorescence. Whereas αSMA expression was highly induced by secreted factors from all MPN BM-MSCs, FN1 production was not induced (Supplementary Fig. 5b, c). This experiment demonstrates that fibrotic BM-MSCs reinforce a fibrotic phenotype of healthy BM-MSCs. Furthermore, medium exchange and coculture experiments indicated that either the regulation of FN1 and αSMA expression is mediated via different pathways/factors or the level of the inducer is different.

After demonstrating the involvement of JAK2/STAT3, TGFβ/SMAD3, and NFκB signaling in the fibrosis induced by BM-MSCs, we wanted to see if treatment of these cells with specific SMAD3 (SIS3), NFκB (JSH23), and JAK1/2 (Ruxo) inhibitors, alone or in combination, could prevent the development of fibrosis in BM-MSCs upon coculture with PB-MNCs with the *JAK2* V617F mutation. We treated the cocultured cells with inhibitors for 48 h and observed the expression of αSMA and FN1 by immunofluorescence. JSH23 and SIS3 concentrations were determined by MTT assay (Supplementary Fig. 5d). To prove that fibrotic cells originate from BM-MSCs and not monocytes present in PB-MNCs, we prestained BM-MSCs with the vital dye carboxyfluorescein succinimidyl ester (CFSE). Coculture of BM-MSCs from healthy donors with PB-MNCs from PMF patients carrying the *JAK2* V617F mutation induced the expression of FN1 and αSMA (Fig. 5b, c). Of note, all cells expressed CFSE, indicating mesenchymal origin (Fig. 5b, c). Treatment with any of the three inhibitors alone led to a significant decrease in FN1 and αSMA expression (Fig. 5b, c, Supplementary Fig. 5e, f). Moreover, inhibition of JAK1/2 and SMAD3 had a synergistic effect in decreasing the expression of FN1 and αSMA, while the NFκB inhibitor displayed an additive effect on αSMA expression in combination with the JAK1/2 inhibitor (Fig. 5b, c, Supplementary Fig. 5e, f). Treatment of fibrosis, induced by *JAK2* V617F-negative PB-MNCs, with SMAD3, NFκB or JAK1/2 inhibitors led to a similar decrease in FN1 and a slightly lower although significant decrease in αSMA expression compared to the respective values in BM-MSCs cocultured with *JAK2* V617F-positive PB-MNCs (Supplementary Fig. 5e–g). Combined JAK1/2 and SMAD3 treatment proved to be the most efficient in preventing the myelofibrosis induced by BM-MSCs via neoplastic MPN mononuclear cells regardless of the presence of *JAK2* mutation.

### Effect of SMAD3, NFκB, and JAK1/2 inhibition on fibrotic mesenchymal stromal cells in MPNs

**Fig. 6:**
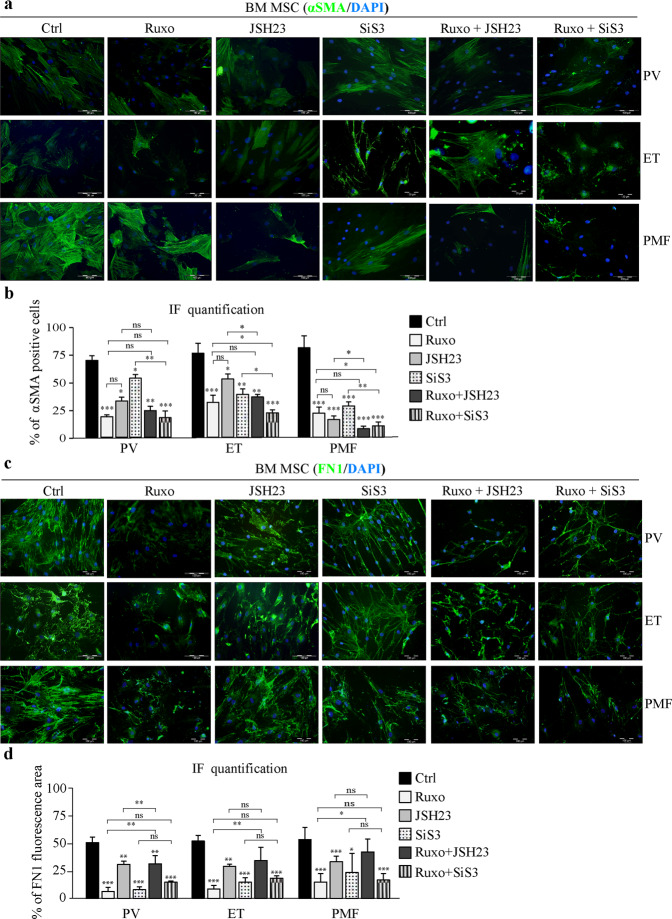
Combined treatment with JAK1/2 and SMAD3 inhibitors decreases fibrosis induced by MPN mesenchymal stromal cells. Bone marrow mesenchymal stromal cells (BM-MSCs) isolated from healthy donors (HDs) or patients with polycythemia vera (PV), essential thrombocythemia (ET) and primary myelofibrosis (PMF) were treated with ruxolitinib (Ruxo) or the SIS3 or JSH23 inhibitor or a combination for 48 h. **a** Immunofluorescence assay for α-smooth muscle actin (αSMA, green). Nuclei were counterstained with 4ʹ,6 diamidino-2-phenylindole (DAPI, blue). Scale bar 100 µm. **b** Percentage of αSMA**-**positive cells. **c** Immunofluorescence assay for fibronectin 1 (FN1, green). Nuclei are counterstained with DAPI (blue). Scale bar 100 µm. **d** Percentage of area with FN1 fluorescence. *n* = 3; **b** and **d** mean ± SEM, *p < 0.05, **p < 0.01, ***p < 0.001.

Since JAK1/2, SMAD3, or NFκB inhibition prevented the development of the BM-MSC fibrotic phenotype, we wanted to test whether it could reduce or resolve the fibrosis induced by MPN BM-MSCs. To this end, we treated BM-MSCs isolated from MPN patients with SIS3, JSH23, or Ruxo alone or in combination for 48 h and observed the expression of αSMA and FN1 by immunofluorescence. Treatment of MPN BM-MSCs with Ruxo led to a significant reduction in αSMA-positive cells (Fig. 6a, b), and the cells had decreased production of FN1 compared to untreated cells (Fig. 6c, d). The NFκB inhibitor JSH23 decreased αSMA expression in MPNs, which was further potentiated by Ruxo in ET and PMF (Fig. 6a, b). JSH23 slightly reduced FN1 production but did not show a synergistic effect in combination with Ruxo (Fig. 6c, d). Treatment of MPN BM-MSCs with the SMAD3 inhibitor SIS3 induced a significant reduction in αSMA-positive cells, while the combination of Ruxo and SIS3 showed even better results in ET and PMF than Ruxo alone (Fig. 6a, b). FN1 production was notably decreased after SIS3 treatment compared to that seen in untreated MPN BM-MSCs, to a level similar to that seen with Ruxo treatment, but combined treatment did not show an additive effect (Fig. 6c, d). These results were corroborated by Western blot analysis (Supplementary Fig. 6a, b). Zymography assays indicated that Ruxo and SIS3 decreased the secretion of profibrotic MMP9, whereas JSH23 led to a slight upregulation of this enzyme (Supplementary Fig. 6c, d). In addition, SIS3 was able to induce a slight increase in the expression of active MMP2, alone or in combination with Ruxo (Supplementary Fig. 6c, d). Together, these results indicate that inhibition of the NFκB and TGFβ/SMAD3 signaling pathways in addition to Ruxo treatment could be beneficial in reversing the fibrotic phenotype of BM-MSCs in MPNs.

## Discussion

Our data demonstrate that BM-MSCs are involved in fibrosis in MPN patients through myofibroblast differentiation, increased ECM production and aberrant ECM degradation. These effects are achieved via activation of and crosstalk between the TGFβ/SMAD3 and JAK1/2/STAT3 pathways and the contribution of inflammatory IL6 and NFκB signaling. The current MPN therapeutic ruxolitinib decreased the fibrosis induced by BM-MSCs isolated from MPN patients, but combined therapy with inhibitors of the TGFβ/SMAD3 signaling pathway had a synergistic effect on fibrosis (Supplementary Fig. 6e). Furthermore, combined ruxolitinib and SMAD3 inhibitor treatment efficiently blocked the development of BM-MSC fibrosis induced by MPN mononuclear cells.

Mononuclear cells from MPN patients were able to induce fibrosis via BM-MSCs independently of the presence or absence of the *JAK2* V617F mutation. Other authors also reported that no significant differences were detected in reticulin and collagen fibrosis between *JAK2* mutation-positive (*JAK2* V617F) and *JAK2* mutation-negative (*CALR* type 1 and type 2, *MPL* or triple negative mutant) mutational subgroups^26–28^. Recent gene expression profiling analysis by Wong et al. showed that the vast majority of genes differentially altered in the bone marrow of fibrotic MPN patients compared to nonfibrotic patients (121/123), while did not show correlation with distinct driver mutations^29^. However, nondriver mutations in the additional sex combs like 1 (ASXL1) and enhancer of zeste homolog 2 (EZH2) genes were reported to be enriched in overtly fibrotic PMF patients^27,28^. These results, together with our findings, indicate that fibrosis in MPNs converges on a common JAK/STAT axis and may be enhanced by additional factors, such as a lack of epigenetic chromatin modifiers or activation of inflammatory pathways.

Although fibrosis was considered to be irreversible until recently, clinical patient follow-up revealed that long-term Ruxo therapy may reverse or markedly delay the progression of BM fibrosis based on changes in reticulin fibrosis grade^30,31^. Our study supports clinical observations, since Ruxo treatment of fibrotic BM-MSCs from MPN patients led to a significant decrease in αSMA expression and FN1 production. Moreover, Ruxo was able to markedly decrease the fibrotic phenotype of BM-MSCs induced by coculture with PB-MNCs, isolated from MPN patients, regardless of their *JAK2* mutational status. Nevertheless, an optimal effect on preventing fibrosis of BM-MSCs by combined Ruxo and SIS3 treatment was observed, offering a novel combination therapy approach.

TGFβ increased pSTAT3 and increased the fibrosis induced by BM-MSCs isolated from healthy donors, which could be abolished if the cells were cotreated with Ruxo, indicating convergence of TGFβ/SMAD and JAK1/2/STAT3 signaling. Moreover, combined inhibition of JAK1/2 and SMAD3 synergistically prevented the development of fibrosis or reversed the BM-MSC fibrotic phenotype in MPN patients. The STAT3 inhibitor Stattic was also able to decrease fibrosis in BM-MSCs from MPN patients (data not shown), but further experiments are needed to assess its effect on TGFβ-induced fibrosis in the absence of aberrantly active JAK/STAT signaling. The involvement of STAT3 in TGFβ cellular effects during fibrogenesis has already been reported in liver fibrosis^32^. In this model, STAT3 activation in the early phase is achieved via JAK1, whereas in the late phase, it requires SMAD3 and TβRI kinase activity^33^. Therefore, it is reasonable to assume that TGFβ-induced BMF largely depends on the balance and integration of the SMAD3 and STAT3 signaling pathways.

Since MMPs degrade ECM, it might be expected that their function is to resolve the excess matrix and decrease fibrosis, with downregulated levels in MPN compared to healthy tissue. However, some MMPs are antifibrotic, whereas others can have profibrotic functions^20^. This notion can be explained by the fact that MMPs participate in a range of other processes, such as cell migration, leukocyte activation, and chemokine processing^34,35^, that can contribute to fibrosis. Previous reports suggest that MMP2 exerts antifibrotic effects in the liver and kidney by influencing the degree of collagen deposition^20^. MMP-9 expression levels correlate positively with the severity of experimental fibrosis^36^. In addition, MMP9 has been shown to activate latent TGFβ1^37^, suggesting that it has a profibrotic role in the lung and kidney. These data are consistent with our findings that the level of active MMP2 decreases in BM-MMPs after TGFβ stimulation. Ruxolitinib treatment, which diminishes the fibrotic phenotype of BM-MSCs, increases secreted MMP2 levels. Profibrotic MMP9 is not detectable in healthy BM-MSCs but is highly upregulated in BM-MSCs, which may reflect the fibrotic status of patients with MPNs.

An increasing body of evidence shows that transplantation of healthy BM-MSCs can successfully reduce hepatic and pulmonary fibrosis^38–40^ due to their anti-inflammatory properties^41^. However, certain growth factors, cytokines, and inflammatory signals in the pathological microenvironment cause BM-MSCs to lose immunomodulatory functions and differentiate into myofibroblasts. This phenomenon was observed in MPN patients in our study, but it has also been reported in other pathological conditions, such as fungal lung infection^42^. Our study showed that in MPNs, IL6 contributes to the fibrotic phenotype of BM-MSCs by affecting αSMA and FN1 levels and decreasing the active form of antifibrotic MMP2. This result is in accordance with the known literature, demonstrating that blockade of IL6 trans-signaling reduces renal fibrosis by decreasing STAT3 phosphorylation^43^. Similar to the case in renal fibrosis, in our model system, it is most likely that IL6, along with other proinflammatory cytokines, boosted JAK/STAT signaling, which cooperated with the TGFβ/SMAD pathway to regulate the expression of fibrotic genes. Waterman and coworkers offered an interesting paradigm in which MSCs can be polarized to either a proinflammatory or immunosuppressive phenotype by priming of different Toll-like receptors^44^. It is possible that similar mechanisms regulate the profibrotic and antifibrotic effects of BM-MSCs, but further research is required to confirm this hypothesis.

At present, the only curative treatment for BMF is allogenic stem cell transplantation, which is able to restore BM function but carries significant morbidity and mortality risks^45^. Thus, finding novel treatment options that target fibrogenic players alone or in combination with a JAK2 inhibitor is an active area of research. Several agents expected to target the BM microenvironment, including immunomodulatory drugs (thalidomide and lenalidomide), proteasome inhibitors (bortezomib), and VEGF-targeting drugs (sunitinib and bevacizumab), have been tested on PMF patients in clinical trials but have shown inconsistent results and severe tolerability issues^46^. Pirfenidone, an inhibitor of fibrogenic cytokines, including platelet-derived growth factor, tumor necrosis factor-α and TGF-β^47^, and a monoclonal antibody antagonizing TGFβ^48^ did not improve BMF. A recent study showed that a Gli inhibitor could attenuate fibrosis by inhibiting BM-MSC expansion and myofibroblast differentiation^17^. Combining sonidegib, a hedgehog inhibitor, with ruxolitinib in a murine transplant model of ET/MF resulted in a significant reduction in BMF^49^. The specific effect of proinflammatory signaling on BMF has yet to be fully evaluated in future studies.

In conclusion, inflammation potentiates TGFβ-induced fibrosis of BM-MSCs via a molecular mechanism involving the SMAD3 and JAK1/2 signaling pathways. Combined inhibition of these signaling pathways can prevent the onset of fibrosis or impair its progression in MPN. Our study reveals the cellular origin and molecular basis of fibrosis, suggesting a novel treatment strategy to reduce morbidity and mortality in PMF and prevent fibrosis progression in PV and ET. Ultimately, strategies aimed at preventing inflammation-induced profibrotic phenotypes may help restore normal hematopoiesis and disrupt the self-reinforcing malignant niche.

## Supplementary information

Supplementary Information File

## References

[1] Jones AV, Cross NC (2013). Inherited predisposition to myeloproliferative neoplasms. Ther. Adv. Hematol..

[2] Viny AD, Levine RL (2014). Genetics of myeloproliferative neoplasms. Cancer J..

[3] Nangalia J, Green AR (2017). Myeloproliferative neoplasms: from origins to outcomes. Blood.

[4] Zahr AA (2016). Bone marrow fibrosis in myelofibrosis: pathogenesis, prognosis and targeted strategies. Haematologica.

[5] Wong WJ (2019). Gene expression profiling distinguishes prefibrotic from overtly fibrotic myeloproliferative neoplasms and identifies disease subsets with distinct inflammatory signatures. PLoS ONE.

[6] Kröger N (2014). Dynamic of bone marrow fibrosis regression predicts survival after allogeneic stem cell transplantation for myelofibrosis. Biol. Blood Marrow Transpl..

[7] Lekovic D (2014). Contribution of comorbidities and grade of bone marrow fibrosis to the prognosis of survival in patients with primary myelofibrosis. Med. Oncol..

[8] Gleitz HFE, Pritchard JE, Kramann R, Schneider RK (2019). Fibrosis driving myofibroblast precursors in MPN and new therapeutic pathways. HemaSphere.

[9] Massaro F, Molica M, Breccia M (2017). How Ruxolitinib modified the outcome in myelofibrosis: focus on overall survival, allele burden reduction and fibrosis changes. Expert. Rev. Hematol..

[10] Agarwal A (2016). Bone marrow fibrosis in primary myelofibrosis: pathogenic mechanisms and the role of TGF-β. Stem Cell Investig..

[11] Popova AP (2010). Autocrine production of TGF-beta1 promotes myofibroblastic differentiation of neonatal lung mesenchymal stem cells. Am. J. Physiol. Lung Cell. Mol. Physiol..

[12] Lecarpentier Y (2018). Human bone marrow contains mesenchymal stromal stem cells that differentiate in vitro into contractile myofibroblasts controlling T lymphocyte proliferation. Stem Cells Int..

[13] Verstovsek S (2016). Role of neoplastic monocyte-derived fibrocytes in primary myelofibrosis. J. Exp. Med..

[14] Maekawa T (2019). Increased SLAMF7high monocytes in myelofibrosis patients harboring JAK2V617F provide a therapeutic target of elotuzumab. Blood.

[15] El Agha E (2017). Mesenchymal stem cells in fibrotic disease. Cell Stem Cell.

[16] El Agha E (2017). Two-way conversion between lipogenic and myogenic fibroblastic phenotypes marks the progression and resolution of lung fibrosis. Cell Stem Cell.

[17] Schneider RK (2017). Gli1+ Mesenchymal stromal cells are a key driver of bone marrow fibrosis and an important cellular therapeutic target. Cell Stem Cell.

[18] Santibáñez JF, Guerrero J, Quintanilla M, Fabra A, Martínez J (2002). Transforming growth factor-beta1 modulates matrix metalloproteinase-9 production through the Ras/MAPK signaling pathway in transformed keratinocytes. Biochem. Biophys. Res. Commun..

[19] Schneider RK (2014). Activated fibronectin-secretory phenotype of mesenchymal stromal cells in pre-fibrotic myeloproliferative neoplasms. J. Hematol. Oncol..

[20] Giannandrea M, Parks WC (2014). Diverse functions of matrix metalloproteinases during fibrosis. Dis. Model. Mech..

[21] Hoermann G, Greiner G, Valent P (2015). Cytokine regulation of microenvironmental cells in myeloproliferative neoplasms. Mediators Inflamm..

[22] Čokić VP (2015). Microarray and proteomic analyses of myeloproliferative neoplasms with a highlight on the mTOR signaling pathway. PLoS ONE.

[23] Shilling AD (2010). Metabolism, excretion, and pharmacokinetics of [14C] INCB018424, a selective Janus tyrosine kinase 1/2 inhibitor, in humans. Drug Metab. Dispos..

[24] Luedde T, Schwabe RF (2011). NF-κB in the liver-linking injury, fibrosis and hepatocellular carcinoma. Nat. Rev. Gastroenterol. Hepatol..

[25] Mercier F, Monczak Y, Francois M, Prchal J, Galipeau J (2009). Bone marrow mesenchymal stromal cells of patients with myeloproliferative disorders do not carry the JAK2-V617F mutation. Exp. Hematol..

[26] Rumi E (2014). Clinical effect of driver mutations of JAK2, CALR, or MPL in primary myelofibrosis. Blood.

[27] Guglielmelli P (2017). Presentation and outcome of patients with 2016 WHO diagnosis of prefibrotic and overt primary myelofibrosis. Blood.

[28] Wong WJ (2018). JAK2, CALR, MPL and ASXL1 mutational status correlates with distinct histological features in Philadelphia chromosome-negative myeloproliferative neoplasms. Haematologica.

[29] Wong WJ (2019). Gene expression profiling distinguishes prefibrotic from overtly fibrotic myeloproliferative neoplasms and identifies disease subsets with distinct inflammatory signatures. PLoS ONE.

[30] Wilkins BS (2013). Resolution of bone marrow fibrosis in a patient receiving JAK1/JAK2 inhibitor treatment with Ruxolitinib. Haematologica.

[31] Kvasnicka HM (2018). Long-term effects of Ruxolitinib versus best available therapy on bone marrow fibrosis in patients with myelofibrosis. J. Hematol. Oncol..

[32] Liu Y (2013). Transforming growth factor-β (TGF-β)-mediated connective tissue growth factor (CTGF) expression in hepatic stellate cells requires Stat3 signaling activation. J. Biol. Chem..

[33] Tang LY (2017). Transforming growth factor-β (TGF-β) directly activates the JAK1-STAT3 axis to induce hepatic fibrosis in coordination with the SMAD pathway. J. Biol. Chem..

[34] Manicone AM, McGuire JK (2008). Matrix metalloproteinases as modulators of inflammation. Semin. Cell Dev. Biol..

[35] Parks WC, Wilson CL, López-Boado YS (2004). Matrix metalloproteinases as modulators of inflammation and innate immunity. Nat. Rev. Immunol..

[36] Murthy S, Ryan A, He C, Mallampalli RK, Carter AB (2010). Rac1-mediated mitochondrial H_2_O_2_ generation regulates MMP-9 gene expression in macrophages via inhibition of SP-1 and AP-1. J. Biol. Chem..

[37] Yu Q, Stamenkovic I (2000). Cell surface-localized matrix metalloproteinase-9 proteolytically activates TGF-beta and promotes tumor invasion and angiogenesis. Genes Dev..

[38] Farouk S, Sabet S, Abu Zahra FA, El-Ghor AA (2018). Bone marrow derived-mesenchymal stem cells downregulate IL17A dependent IL6/STAT3 signaling pathway in CCl4-induced rat liver fibrosis. PLoS ONE.

[39] Luo XY (2019). Transplantation of bone marrow mesenchymal stromal cells attenuates liver fibrosis in mice by regulating macrophage subtypes. Stem Cell Res. Ther..

[40] Chen J, Si L, Zhou L, Deng Y (2019). Role of bone marrow mesenchymal stem cells in the development of PQ‑induced pulmonary fibrosis. Mol. Med. Rep..

[41] Regulski MJ (2017). Mesenchymal stem cells: “guardians of inflammation”. Wounds.

[42] Arango JC (2017). Impaired anti-fibrotic effect of bone marrow-derived mesenchymal stem cell in a mouse model of pulmonary paracoccidioidomycosis. PLoS Negl. Trop. Dis..

[43] Chen W (2019). Blocking interleukin-6 trans-signaling protects against renal fibrosis by suppressing STAT3 activation. Theranostics.

[44] Waterman RS, Tomchuck SL, Henkle SL, Betancourt AM (2010). A new mesenchymal stem cell (MSC) paradigm: polarization into a pro-inflammatory MSC1 or an immunosuppressive MSC2 phenotype. PLoS ONE.

[45] Kröger N (2007). Rapid regression of bone marrow fibrosis after dose-reduced allogeneic stem cell transplantation in patients with primary myelofibrosis. Exp. Hematol..

[46] Rambaldi A, Barbui T, Barosi G (2008). From palliation to epigenetic therapy in myelofibrosis. Hematol. Am. Soc. Hematol. Educ. Program.

[47] Mesa RA (2001). A phase II trial of pirfenidone (5-methyl-1-phenyl-2-[1H]-pyridone), a novel anti-fibrosing agent, in myelofibrosis with myeloid metaplasia. Br. J. Haematol..

[48] Mascarenhas J (2014). Antitransforming growth factor beta (TGF-β) therapy in patients with myelofibrosis. Leuk. Lymphoma.

[49] Gupta V (2020). Safety and efficacy of the combination of sonidegib and ruxolitinib in myelofibrosis: a phase 1b/2 dose-finding study. Blood Adv..

