# Supplementary material for: Inhibition of proinflammatory signaling impairs fibrosis of bone marrow mesenchymal stromal cells in myeloproliferative neoplasms
Source: Exp Mol Med. 2022 Mar 14;54(3):273–84. doi: 10.1038/s12276-022-00742-y (PMC8980093; doi:10.1038/s12276-022-00742-y)
Supplement: Supplementary file 1 — Supplementary Information File [file 12276_2022_742_MOESM1_ESM.pdf]

Supplementary Information File

Inhibition of proinflammatory signaling impairs fibrosis of bone marrow mesenchymal stromal cells in myeloproliferative neoplasms

Milica Vukotić, Sunčica Bjelica, Teodora Dragojević, Dragoslava Đikić, Olivera Mitrović Ajtić, Miloš Diklić, Tijana Subotički, Emilija Živković, Bojana Beleslin Čokić, Aleksandar Vojvodić, Juan F. Santibanez, Mirjana Gotić, Vladan P. Čokić

Supplementary Information File contains:

- **Supplementary Figures and Figure Legends 1-6**
- **Supplementary Methods**

Supplementary Fig. 1.

a

Diagnosis	Age	Gender	JAK2 mutation	Gradus of fibrosis
**PMF**	78	F	+	0
**PV**	80	M	+	1
**PMF**	67	M	+	3
**PV**	61	M	+	1
**PV**	63	M	+	0
**PMF**	71	M	+	0
**PV**	78	M	+	0
**PMF**	70	F	+	0
**PMF**	67	M	+	1
**PV**	65	M	+	0
**ET**	55	M	+	0
**PMF**	74	M	+	1
**ET**	64	F	+	1
**PMF**	64	F	+	2

Diagnosis	Age	Gender	JAK2 mutation	Gradus of fibrosis
**PMF**	82	F	+	1
**PV**	83	F	+	0
**PV**	66	F	+	0
**PV**	74	M	+	0
**ET**	59	F	-	1
**ET**	31	F	+	0
**ET**	82	F	+	1
**PMF**	88	M	-	1
**PV**	74	F	+	0
**PV**	40	M	+	0
**PV**	63	F	+	0
**ET**	70	M	+	1
**ET**	33	M	+	0

b

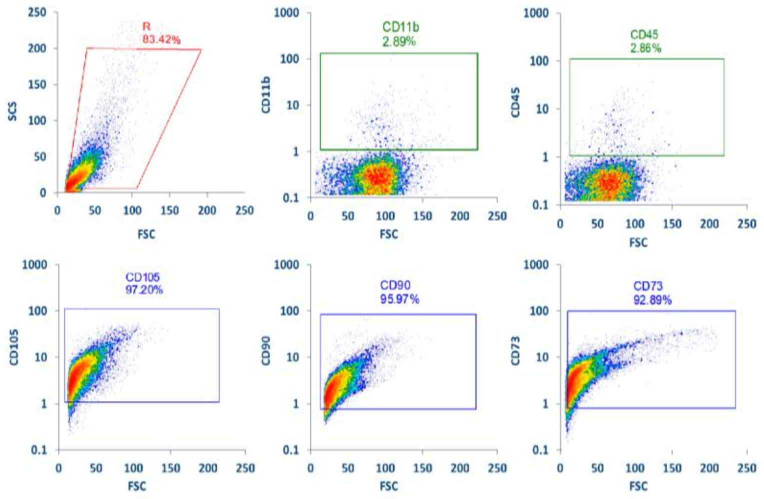

Surface marker	% of positive cells
**CD105**	97,20
**CD90**	95,97
**CD73**	92,89
**CD11b**	2,89
**CD45**	2,86

c

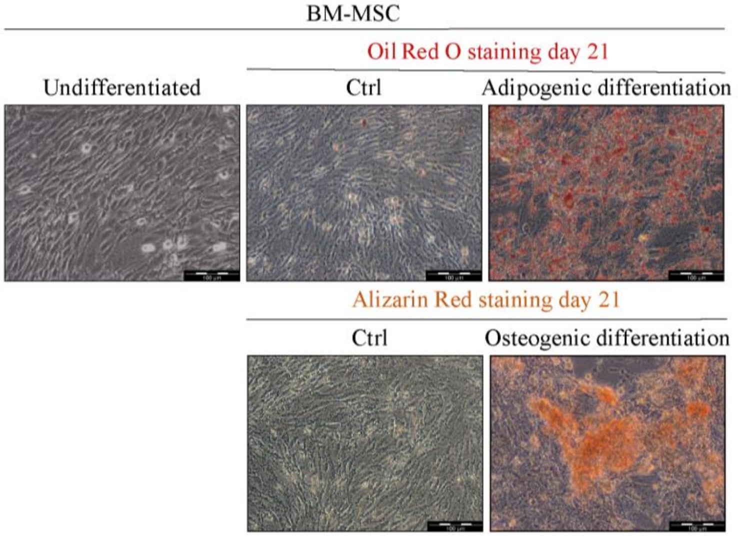

Supplementary Fig. 1. Bone marrow mesenchymal stromal cells from MPN patients display fibrotic phenotype. Bone marrow mesenchymal stromal cells **(**BM-MSC) isolated from healthy donors (HD), or patients with polycythemia vera (PV), essential thrombocythemia (ET) and primary myelofibrosis (PMF). **a)** Table representing characteristics of patients whose BM-MSC are used in this study. **b)** Expression of BM-MSC surface markers was tested by flow cytometry. Representative gating images (left) and percentage of CD73-, CD90-, and CD105-positive and CD11b- and CD45-negative cells (right). **c)** Fibroblast-like morphology of undifferentiated BM-MSC. BM-MSC differentiate into adipocytes and osteocytes upon induction in appropriate medium for 21 days. Adipocytes were stained with Oil Red O, while osteocytes were stained with Alizarin Red (n = 4).

Supplementary Fig. 2.

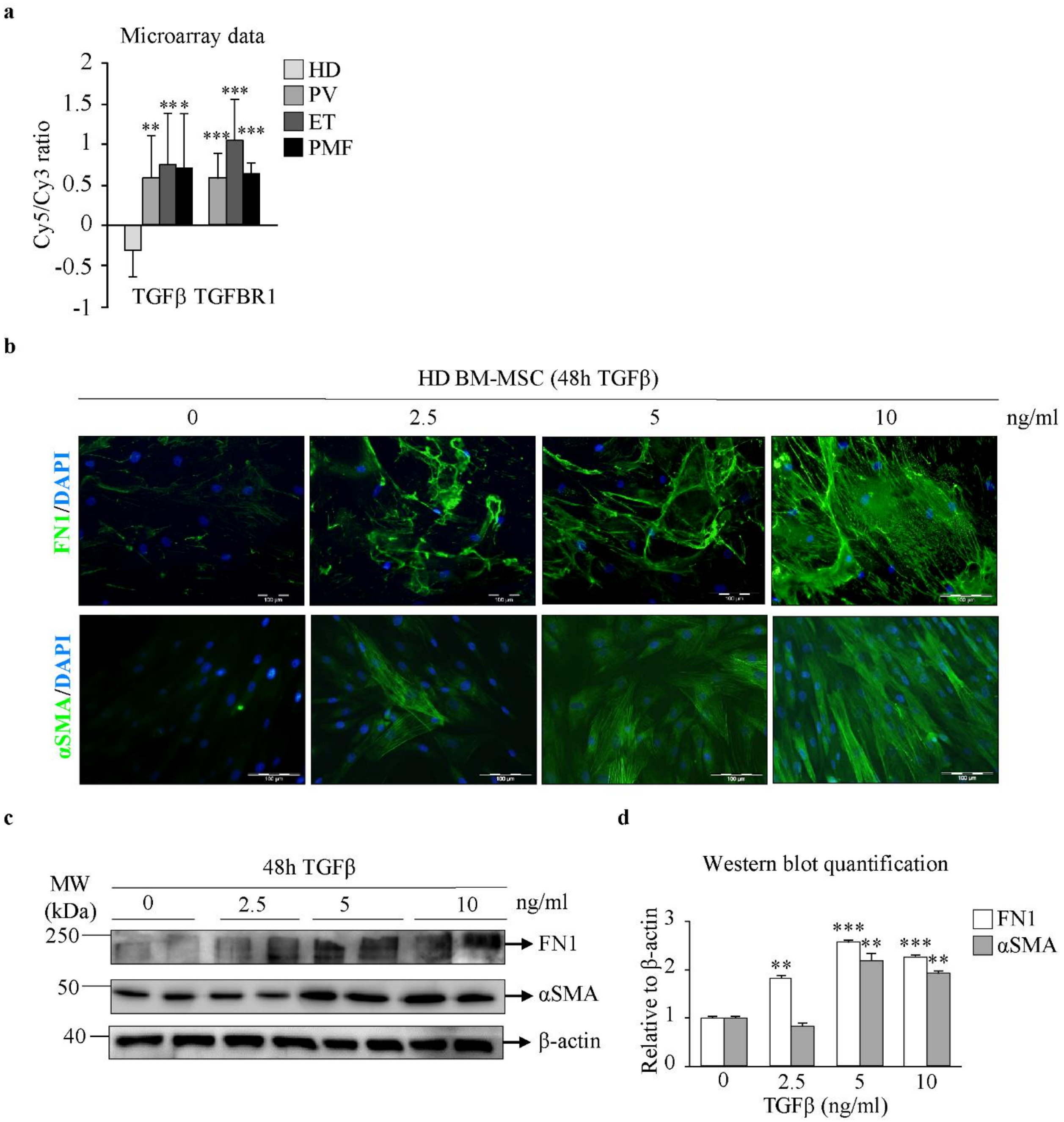

Supplementary Fig. 2. TGFβ and JAK2/STAT3 signaling in bone marrow mesenchymal stromal cells from MPN patients a) CD34-positive cells isolated from peripheral blood of patients with PV, ET, and PMF, as well as HD. Microarray data indicating mRNA expression levels of TGFβ and TGFBR1 comparing to human universal RNA (huRNA). **b)** Healthy bone marrow mesenchymal stromal cells **(**HD BM-MSC) were treated with indicated concentrations of transforming growth factor β (TGFβ) for 48h. Immunofluorescence assays for fibronectin (FN1) and α-smooth muscle actin (αSMA, green). Nuclei were counterstained with 4 6

diamidino-2-phenylindole (DAPI, blue). Scale bar 100µm. **c)** Western blot indicating protein expression of αSMA and FN1. β-actin was used as a loading control. **d)** Quantification of αSMA and FN1 Western blot bands. n = 4; a) and d) mean + SEM, *p < 0.05, **p < 0.01, ***p < 0.001.

Supplementary Fig. 3.

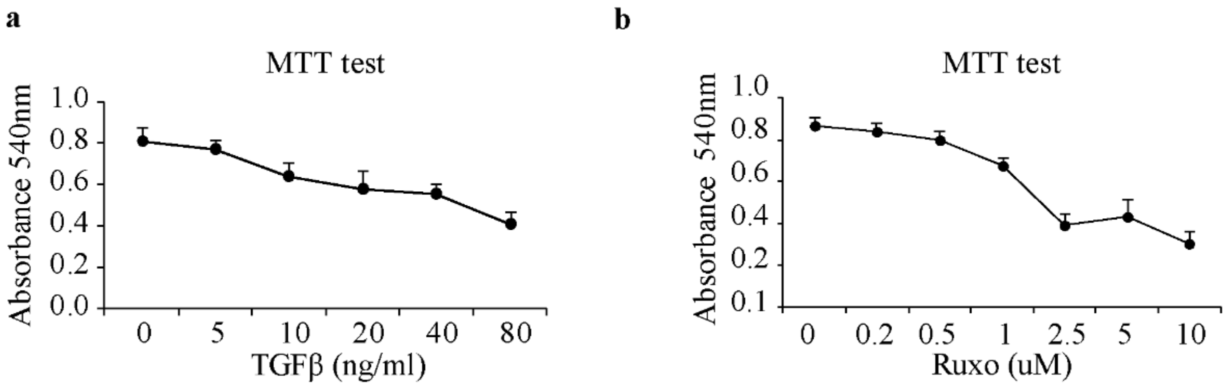

Supplementary Fig. 3. Treatment with Ruxolitinib decreases TGFβ-induced fibrosis in mesenchymal stromal cells. MTT test indicating absorbance at 540nm (y axis) after treatment of bone marrow mesenchymal stromal cells with indicated concentrations of: **a)** transforming growth factor β (TGFβ, x axis). and **b)** Ruxolitinib **(**Ruxo, x axis).

Supplementary Fig. 4.

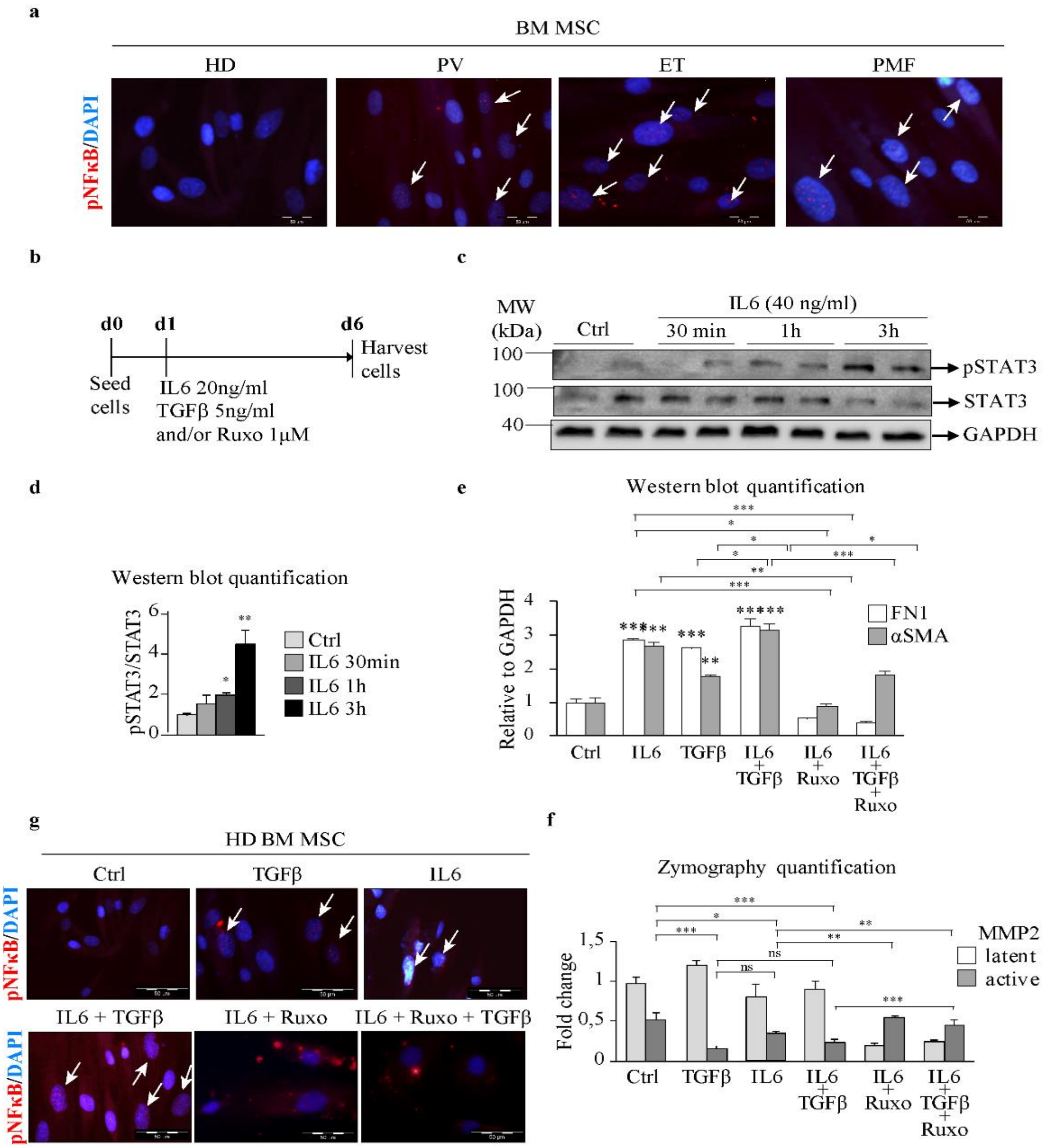

Supplementary Fig. 4. Inflammatory IL6 signaling contributes to TGFβ-induced fibrosis in mesenchymal stromal cells. a) Immunofluorescence assay for phosphorylated nuclear factor kappa-light-chain-enhancer of activated B cells (pNFκB) (red) on BM-MSC cells isolated from healthy donors (HD) and patients with polycythemia vera (PV), essential thrombocythemia (ET) and primary myelofibrosis (PMF). Nuclei were counterstained with 4 6 diamidino-2-phenylindole (DAPI, blue). Scale bar 50µm. Arrows indicate nuclear localization of pNFκB. **b)**

HD BM-MSC cells were treated with indicated concentrations of interleukin 6 (IL6) and transforming growth factor beta (TGFβ) as depicted on the scheme. **c)** HD BM-MSC cells were treated with 40ng/ml IL6 for 30 min, 1h or 3h. Western blot indicating protein expression of phosphorylated signal transducer and activator of transcription 3 (pSTAT3) and total STAT3. Glyceraldehyde 3-phosphate dehydrogenase (GAPDH) was used as a loading control. **d)** Quantification of STAT3 Western blot bands. Further on, HD BM-MSC cells were treated with IL6, TGFβ, and/or Ruxolitinib (Ruxo). **e)** Quantification of αSMA and FN1 Western blot bands in Figure 5F. **f)** Quantification of MMP2zymography assay bands depicted in Figure 5G. **g)** Immunofluorescence assay for pNFκB (red). Nuclei were counterstained with 4 6 diamidino-2-phenylindole (DAPI, blue). Scale bar 50μm. Arrows indicate nuclear localization of pNFκB. $n = 4$; d-f) mean + SEM, *$p < 0.05$, **$p < 0.01$, ***$p < 0.001$.

Supplementary Fig. 5.

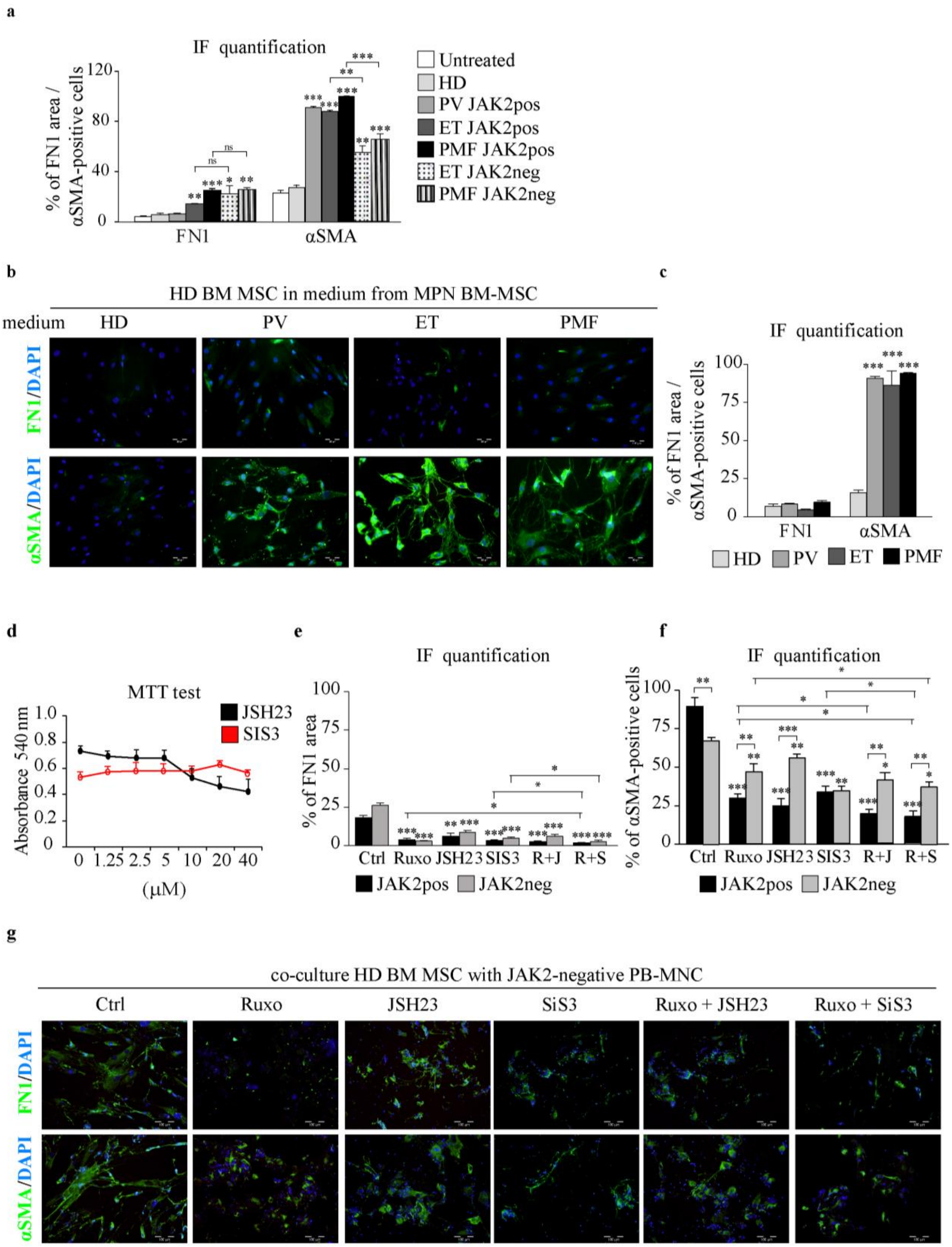

Supplementary Fig. 5. MPN mononuclear cells induce fibrosis in mesenchymal stromal cells. a) Quantification of the area of fibronectin 1 (FN1) and percentage of α-smooth muscle

actin (αSMA)-positive cells from Figure 5a. **b)** Healthy BM-MSC grown in medium from fibrotic BM-MCS of PV, ET and PMF patients. Immunofluorescence assay of FN1 and αSMA shown in green. Nuclei are counterstained with 4 6 diamidino-2-phenylindole (DAPI, blue). Scale bar 100µm. **c)** Quantification of the area of FN1 and percentage of αSMA -positive cells. **d)** MTT test indicating absorbance at 540nm (y axis) after treatment of cells with indicated concentrations (x axis) of NFκB inhibitor JSH23 (black line) or SMAD3 inhibitor SIS3 (red line). **e)** Quantification of the area of FN1, and **f)** percentage of αSMA-positive cells from Figure 5b, 5c, and Supplementary Figure 5g. **g)** Healthy BM-MSC incubated with PB-MNC isolated from JAK2-negative PMF patients and treated with specific SMAD3 (SIS3), NFκB (JSH23) and JAK1/2 (Ruxo) inhibitors, alone or in combination for 48h. Immunofluorescence of FN1 and αSMA shown in green. Nuclei are counterstained with DAPI (blue). Scale bar 100µm. n=3, b), d), and g): mean + SEM, $^*p < 0.05$, $^{**}p < 0.01$, $^{***}p < 0.001$.

Supplementary Fig. 6.

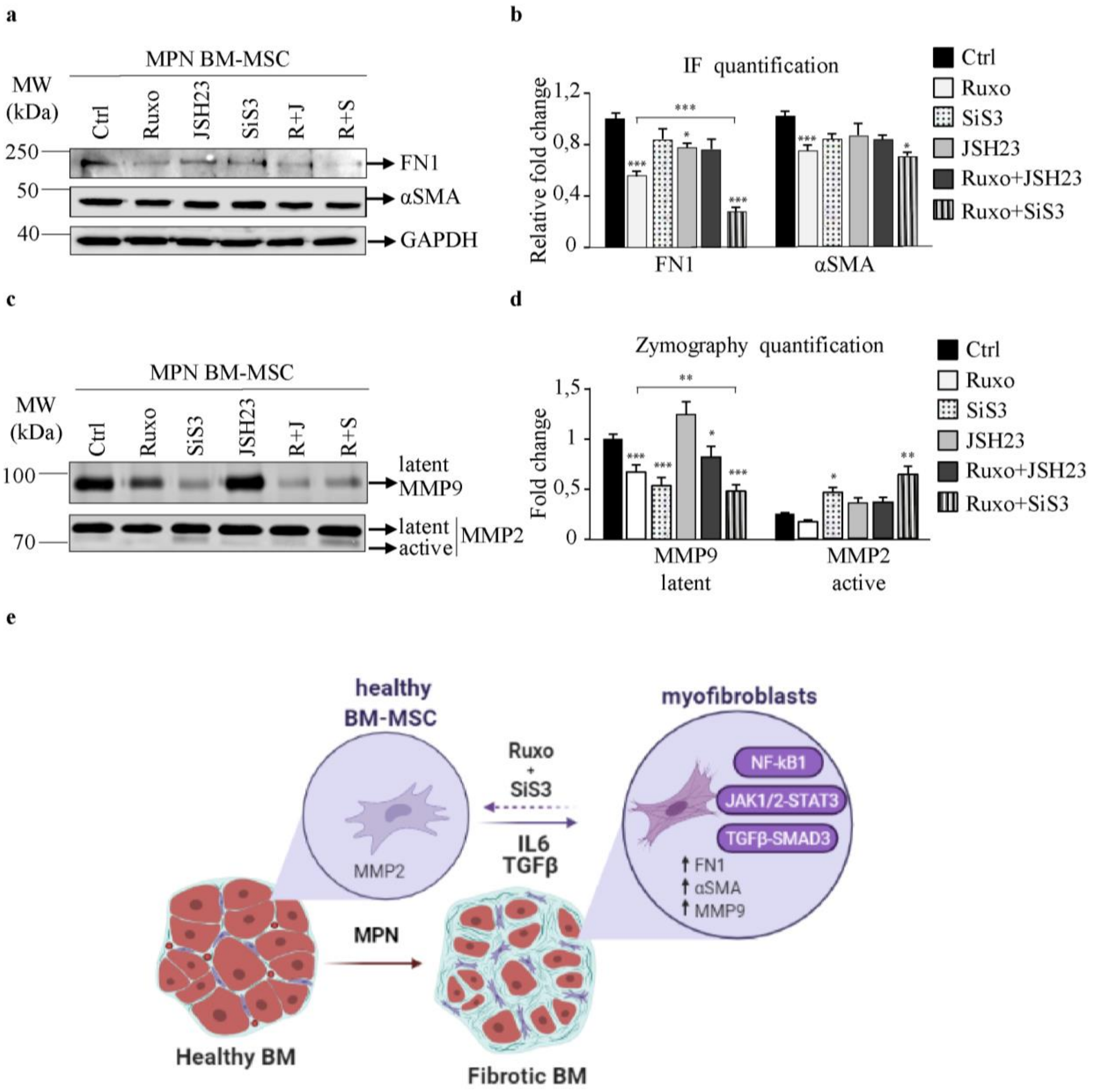

Supplementary Fig. 6. Combined treatment with JAK1/2 and SMAD3 inhibitors decreases fibrosis of MPN mesenchymal stromal cells. Bone marrow mesenchymal stromal cells (BM-MSC) isolated from healthy donors (HD), or patients with polycythemia vera (PV), essential thrombocythemia (ET) and primary myelofibrosis (PMF) were treated with Ruxolitinib (Ruxo), SIS3 or JSH23 inhibitors or combination for 48h. **a)** Western blot indicating protein expression of fibronectin 1 (FN1) and α-smooth muscle actin (αSMA). Glyceraldehyde 3-phosphate dehydrogenase (GAPDH) was used as a loading control. **b)** Quantification of FN1 and αSMA Western blot bands. **c)** Zymography assay showing levels of latent and active form of secreted matrix metalloproteinase 2 and 9 (MMP2 and MMP9). **d)** Quantification of MMP2 and MMP9

zymography assay bands. $n = 3$; D) and F) mean + SEM, $*p < 0.05$, $**p < 0.01$, $***p < 0.001$. **e)** Scheme depicting summarized findings of this study. BM-MSC from MPN patients display myofibrotic phenotype characterized by the expression of αSMA, FN1 and MMP9. Proinflammatory NFκB and JAK2/STAT3 and fibrogenic TGFβ/SMAD3 signaling pathways are activated in MPN BM-MSC. Treatment of MPN BM-MSC with JAK1/2, NFκB or SMAD3 inhibitor decreases αSMA positivity and FN1 production. Combined treatment of Ruxo and SIS3 displays synergistic effect on αSMA expression in ET and PMF.

Supplementary methods

Bone marrow mesenchymal stromal cell culture

Bone marrow aspirates from 27 newly diagnosed MPN patients were obtained from Clinic of Hematology, Clinical Center of Serbia. Bone marrow from 8 healthy donors was obtained during hip replacement surgery at the Department of Orthopedic Surgery, Clinical Center Zemun. All of the donors signed the consent form approved by a local ethical committee in accordance with the Declaration of Helsinki. Mononuclear cells were obtained by density gradient centrifugation on lymphocyte separation media (Capricorn Scientific, Ebsdorfergrund, Germany). Cells were plated at density of $2\times10^5/cm^2$ in cell culture flasks, and cultured with Dulbecco's modified Eagle's medium (DMEM, Sigma-Aldrich, St. Louis, Missouri, USA) supplemented with 10% FBS and 100 units/mL Penicillin/Streptomycin (Biowest, Nuaillé, France) in a humidified atmosphere at 37°C with 5% CO_2. After 2-3 weeks, adherent colonies with fibroblast-like morphology became visible. Then, cells were plated for 3 passages and cultured in the above-mentioned conditions to obtain homogenous cell culture population.

PB-MNC isolation and culture

PB-MNC were isolated from 9 MPN patients, 3 of each diagnosis. Blood was collected in tubes with ethylenediaminetetraacetic acid (EDTA, Sigma-Aldrich) and separated in Lymphocyte cell separation media by gradient centrifugation. The PB-MNC-containing buffy coat was carefully retrieved, washed with phosphate buffered saline (PBS), and incubated in erythrocyte lysis buffer (8,025g NH4Cl, 1,008g NaHCO3, 0,37g EDTA) followed by 10-minute centrifugation. The obtained PB-MNC pellet was resuspended in RPMI-1640 medium (Biowest) and supplemented with 10% FBS, 100 units/mL Penicillin/Streptomycin.

Microarray gene expression analysis

For the assessment of mRNA expression levels in circulatory $CD34^+$ cells biological replicates of nine healthy donors, seven PV patients, nine ET patients, and four PMF patients were analyzed. The RNA samples were processed and analyzed using total human universal RNA (HuURNA, BD Biosciences, Franklin Lakes, New Jersey, USA) as a reference in the

competitive hybridizations as reported before[19]. The microarray data obtained are available at the Gene Expression Omnibus (http://www.ncbi.nlm.nih.gov/geo; accession no. GSE55976).

Flow cytometry

To confirm mesenchymal stromal cell identity, expression of cell surface antigens was analyzed by flow cytometry. Cells were fixed in formaldehyde and stained with fluorescein isothiocyanate (FITC)- or phycoerythrin (PE)-conjugated antibodies directed against human CD73-PE (BioLegend, San Diego, California, USA, 127205), CD90-PE (BioLegend, 328109), CD105-PE (eBioscience, San Diego, California, USA, 12-4714-81), CD11b-FITC (eBioscience, 11-0118-42) and CD45-FITC (R&D Systems, Minneapolis, Minnesota, USA, FAB114F). Stained cells were analyzed by flow cytometry using BD FACS Calibur (BD Bioscience). For each sample, 10000 events were recorded.

Cell differentiation

For osteogenic differentiation, cells were cultured in DMEM supplemented with 10% FBS, 1% penicillin/streptomycin, 10mM β-glycerophosphate, 10nm dexamethasone, and 50 μg/ml vitamin C for 21 days. Adipogenesis was induced by adipogenic medium (DMEM supplemented with 10% FBS, 1% penicillin/streptomycin, 0.5mM isobutylmethylxanthine, 125nM indomethacin, 5μM dexamethasone, 850nM insulin, and 1μM rosiglitazone). Seven days later, cells were switched to the maintenance medium containing 10% FBS, 850nM insulin, and 1μM rosiglitazone. Cells were harvested and analyzed 21 days after induction of differentiation. Oil red O solution was prepared by dissolving stock solution (0.5% Oil Red O in isopropanol) in dH_2O 3:2 and filtered. 2% Alizarin Red S was dissolved in dH_2O and filtered. Fixed cells were incubated in dye solution for 30 min and observed under a phase-contrast microscope (Olympus Provis AX70).

MTT test

Cytotoxic effect of various compounds on BM-MSC cells was analyzed by MTT assay as described in Krstic et al., 2015. Briefly, 10^4 cells/well were seeded in 96 well plates. The following day cells were treated with indicated concentrations of TGFβ, Ruxo, JSH23 and SIS3

for 48h. After this period, MTT (Sigma-Aldrich) was added to each well at final concentration of 0.5 mg/ml, and incubated for 2 h. Culture medium was discarded and the cell-precipitated formazan crystals were dissolved in isopropanol:DMSO mixture in the ratio 3:2 and the absorbance was read at 630nm.

Protein isolation and Western blot analysis

Whole cell extract was obtained from cell pellets by extraction in RIPA buffer (1 mM EDTA, 50 mM Tris-HCl pH 7.5, 0.1% SDS, 150 mM NaCl, 1% NP40, 1% Sodium deoxycholate, protease inhibitor cocktail). Homogenates were separated on polyacrylamide gels and blotted to Hybond nitrocellulose membranes (GE Healthcare, Chicago, Illinois, USA). Membranes were decorated using antibodies directed against: SMAD2/3 (Santa Cruz, sc-8332), pSMAD2 Ser423/425 (Cell Signaling, 3101), STAT3 (Cell Signaling, 9132), pSTAT3 Tyr705 (Cell signaling, 9131S), β-actin (R&D Systems, MAB8928), αSMA (Abcam, ab7817), FN1 (Sigma, F7387), pNFκB Ser536 (Cell Signaling, 3031), TSG6 (Santa Cruz, Santa Cruz, California, USA, sc-377277), TGFBR1 (Santa Cruz, sc-518018), and GAPDH (Santa Cruz Biotechnology, sc-32233) and incubated over night at 4°C. Secondary antibodies conjugated to horseradish peroxidase (GE Healthcare) were detected using an enhanced chemiluminescence detection system (BioRad, Hercules, California, USA). Protein band were visualized using ChemiDoc Imager (BioRad) and quantified in ImageLab software.

Azan trichrome staining

Azan trichrome staining was performed using the following procedure: sections were deparaffinised using xylene and hydrated with descending alcohol series (100%, 96% and 70%) each for 15 min. Thereafter, they were rinsed in distilled water and immersed in the azocarmine B solution for 30 min, rinsed two times under running water and once in distilled water. The sections were differentiated in 1% aniline alcohol solution for 1 min; this was followed by one change of acetic alcohol solution to terminate the differentiation. They were immersed in 5% aqueous phosphotungstic acid for 60 min, after which they were immersed for 60 min in aniline blue-orange G and distilled water mixture in 1:3 ratio and rinsed in distilled water. Sample was then taken through an ascending series of alcohol solution (70%, 96% and 100%) to dehydrate the sections for 2 min each. Sections were finally cleared in xylene 2 times for 5 min and

mounted with Canada balsam. Nuclei, erythrocytes, fibrin, acidophilic cytoplasm are stained red with azocarmine. Collagen fibers, arranged in bundles, are stained blue with aniline blue. Images were taken with microscope (Olympus Provis AX70).

Immunohistochemistry

Tissues were fixed in 10% buffered formalin and embedded in paraffin. For immunofluorescence analyses, 5 μm paraffin sections were deparaffinized, rehydrated, and treated with 3% H_2O_2 solution in PBS to block endogenous peroxidase activity. Samples were incubated with primary antibody directed against TGFβ (R&D systems, 240-B-002) or IL6 (Novocastra, Newcastle upon Tyne, United Kingdom, NCL-L-IL6) in a humid chamber overnight at 4°C. Immunostaining was proceeded using the streptavidin–biotin technique (LSAB+/HRP Kit, DAKO, Glostrup, Denmark). Immunoreactivity was visualized with DAKO Liquid DAB^+ Substrate/Chromogen System counterstained with Mayer's hematoxylin (Merck, Kenilworth, New Jersey, United States) and evaluated under a light microscope (Olympus Provis AX70). For negative control samples, normal serum and TBS buffer (1:500) were pipetted without primary antibodies. Quantification of positive cells was performed by counting 5 representative images for each patient and normalizing to the total number of cells.

Statistical analysis

All data were represented as positive standard error of the mean (SEM). Significance was calculated by two-tailed Student's t-test on Fig. 1d, 1f, 2b, 2d, 2f, 2h, 4b, 4d and Supplementary Fig. 2a, 2d, 4d, and 5c. Two-way ANOVA test was used to determine significance on Fig. 3d, 3f, 6b, 6d, and Supplementary Fig. 4e, 4f, 5a, 5e, 5f, 6b, and 6d with p-values *$p < 0.05$, **$p < 0.01$, ***$p < 0.001$.
